# Hypoxic-induced resting ventilatory and circulatory responses under multistep hypoxia is related to decline in peak aerobic capacity in hypoxia

**DOI:** 10.1186/s40101-022-00310-3

**Published:** 2022-10-24

**Authors:** Masahiro Horiuchi, Shohei Dobashi, Masataka Kiuchi, Yoshiyuki Fukuoka, Katsuhiro Koyama

**Affiliations:** 1 Faculty of Sports and Life Science, National Institute of Fitness and Sports in Kanoya, Shiromizu town 1, Kanoya city, Kagoshima, 8912393 Japan; 2 Division of Human Environmental Science, Mt. Fuji Research Institute, Kami-yoshida 5597-1, Fuji-yoshida city, Yamanashi, 4030005 Japan; 3 Graduate School of Education, University of Yamanashi, Takeda 4-4-37, Kofu city, Yamanashi, 4008510 Japan; 4 Graduate School of Health and Sports Science, Juntendo University, Hiraka-gakuendai 1-1, Inzai city, Chiba, 2701695 Japan; 5 Graduate School Department of Interdisciplinary Research, University of Yamanashi, Takeda 4-4-37, Kofu city, Yamanashi, 4008510 Japan; 6 Faculty of Health and Sports Science, Doshisha University, Tatara-miyakodani 1-3, Kyotanabe city, Kyoto, 6100394 Japan; 7 Faculty of Sport Science, Yamanashi Gakuin University, Sakaori 2-4-5, Kofu city, Yamanashi, 4008575 Japan

**Keywords:** Arterial oxygen saturation, Heart rate, Multiple regression analysis, Pulmonary ventilation

## Abstract

**Background:**

Several factors have been shown to contribute to hypoxic-induced declined in aerobic capacity. In the present study, we investigated the effects of resting hypoxic ventilatory and cardiac responses (HVR and HCR) on hypoxic-induced declines in peak oxygen uptake ($\dot{\mathrm V}$O_2peak_).

**Methods:**

Peak oxygen uptakes was measured in normobaric normoxia (room air) and hypoxia (14.1% O_2_) for 10 young healthy men. The resting HVR and HCR were evaluated at multiple steps of hypoxia (1 h at each of 21, 18, 15 and 12% O_2_). Arterial desaturation (ΔSaO_2_) was calculate by the difference between SaO_2_ at normoxia—at each level of hypoxia (%). HVR was calculate by differences in pulmonary ventilation between normoxia and each level of hypoxia against ΔSaO_2_ (L min^−1^ %^−1^ kg^−1^). Similarly, HCR was calculated by differences in heart rate between normoxia and each level of hypoxia against ΔSaO_2_ (beats min^−1^ %^−1^).

**Results:**

$\dot{\mathrm V}$O_2peak_ significantly decreased in hypoxia by 21% on average (*P* < 0.001). HVR was not associated with changes in $\dot{\mathrm V}$O_2peak_. ΔSaO_2_ from normoxia to 18% or 15% O_2_ and HCR between normoxia and 12% O_2_ were associated with changes in $\dot{\mathrm V}$O_2peak_ (*P* < 0.05, respectively). The most optimal model using multiple linear regression analysis found that ΔHCR at 12% O_2_ and ΔSaO_2_ at 15% O_2_ were explanatory variables (adjusted *R*^2^ = 0.580, *P* = 0.02).

**Conclusion:**

These results suggest that arterial desaturation at moderate hypoxia and heart rate responses at severe hypoxia may account for hypoxic-induced declines in peak aerobic capacity, but ventilatory responses may be unrelated.

## Background

Recently, there have been an increase in the numbers of individuals visiting high-altitude locations for work, mountaineering, skiing, or exercise training. Maintaining physical performance, including aerobic capacity, at high altitude may be important for maximizing training adaptation and preventing accidents.

However, it is well known that peak aerobic capacity (i.e., peak oxygen uptake; $\dot{\mathrm V}$O_2peak_) progressively declines with increasing altitude [1]. Since $\dot{\mathrm V}$O_2_ during moderate-intensity exercise is similar between normoxia and hypoxia ~12% O_2_ [2, 3], a decline in $\dot{\mathrm V}$O_2peak_ at high altitude may affect submaximal exercise performance. Nonetheless, the underlying mechanism(s) causing the decline in $\dot{\mathrm V}$O_2peak_ at high altitude are controversial and have no universal consensus.

Hypoxic ventilatory responses (HVRs) can explain the reductions in $\dot{\mathrm V}$O_2peak_ in hypoxia [4, 5]; however, another study disagreed [6]. An important point is that individual-related variability in response to hypoxic exposure may relate to hypoxic-induced changes in peak aerobic capacity [6]. Moreover, these studies evaluated only respiratory responses to hypoxia [4–6] without evaluation of hypoxic cardiac responses to account for declines in $\dot{\mathrm V}$O_2peak_. Previous studies assessed HVR at only one level of hypoxia [5, 6]. To our knowledge, no studies evaluated HVRs at multiple steps of hypoxia against changes in peak aerobic capacity. Additionally, increasing altitude decreased $\dot{\mathrm V}$O_2peak_ [1] and blunted hypoxic cardiac responses (HCR) [7]; however, to the best of our knowledge, no studies have examined about a relationship between changes in $\dot{\mathrm V}$O_2peak_ and HCR even at one level of hypoxia. Thus, the level of hypoxia and resting hypoxic ventilatory and/or cardiac responses are necessary to exert hypoxic-induced changes in peak aerobic capacity effects. A previous study reported that climbers who maintained their arterial oxygen saturation level from an altitude of 2400 m to 5300 m did not develop symptoms of acute mountain sickness [8]. Thus, considering about actual site, progressive declines in oxygen level protocol may be relevant because it could simulate actual mountain climbing such as from sea level to gradual ascent.

This study therefore aimed to evaluate resting hypoxic respiratory and circulatory responses at multiple steps of hypoxia and to explain hypoxic-induced declines in $\dot{\mathrm V}$O_2peak_. We hypothesized that resting hypoxic respiratory and/or circulatory responses may account for declines in $\dot{\mathrm V}$O_2peak_ in hypoxia and that these responses would change with different oxygen levels.

## Method

### Participants

This study used additional data from already published investigations [9] from an entirely different perspective, which was approved by the ethical committee of the Mount Fuji Research Institute in Japan and performed in accordance with the guidelines of the Declaration of Helsinki. Ten healthy male lowlanders were involved with a mean age of 23 ± 2 years, height of 174.3 ± 7.8 cm, and body weight of 72.4 ± 16.7 kg (mean ± standard deviation). The participants did not engage in regular exercise. They were free from any known cardiorespiratory and cardiovascular diseases and had not taken any medications. Since a previous study demonstrated that previous intermittent hypoxic exposure influences hypoxic ventilatory and cardiovascular responses [10], none of the participants was exposed to an altitude higher than 1500 m within 6 months before the study in accordance with a previous study [11]. Participants were requested to abstain from caffeinated beverages for 12 h and from strenuous physical activity and alcohol for at least 24 h before the study. They were familiarized with all measurement techniques, i.e., cycling exercise at 60 revolutions per minute, hypoxic exposure with a face mask. All participants signed an informed consent form.

### Procedures

The study comprised three experimental protocols: determination of $\dot{\mathrm V}$O_2peak_ (i) in normoxia, (ii) in hypoxia and (iii) in progressive normobaric hypoxia. All studies were performed at an ambient temperature of 24 ± 1°C.

A determination of individual $\dot{\mathrm V}$O_2peak_ was performed using a bicycle ergometer (Monark 828E; Monark, Vansbro, Sweden) while inspiring room air (normobaric normoxia) or 14.1% O_2_ (normobaric hypoxia). This inspired oxygen fraction is equivalent to an altitude of 3200 m at which a large drop in oxygen saturation is observed with a small drop in pressure of O_2_ [12], which in turn acts as a stimulator to enhance exercise performance in many athletes engaging in hypoxic training [13]. Participants performed an incremental leg cycling test with an increasing rate of 30 watts per minute at 60 revolutions per minute in an upright position until exhaustion. The criteria for exhaustion were as follows: (i) no increase in oxygen uptake, despite a further increase in work rate; (ii) heart rate 95% of age-predicted maximal values (220—age); (iii) rating of perceived exertion reaching 19; or (iv) failure to maintain pedaling frequency of 60 revolutions per minute despite a strong verbal encouragement. The test was terminated when at least two of the above four criteria were met [14]. The order of these two tests was randomized and counterbalanced.

**Fig. 1:**
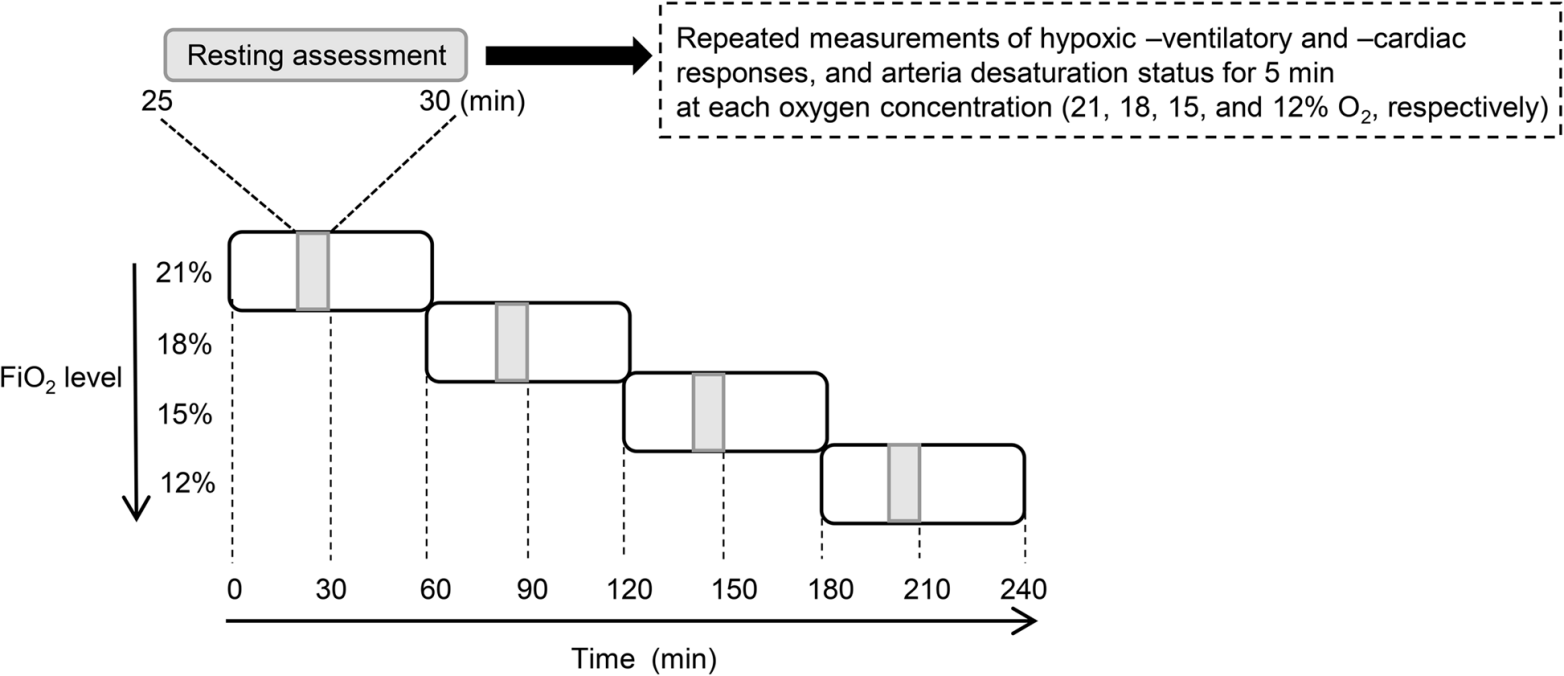
Experimental protocol for the assessment of resting hypoxic, ventilatory, and cardiac responses and arterial desaturation status. FiO_2_, fraction of inspired oxygen

**Fig. 2:**
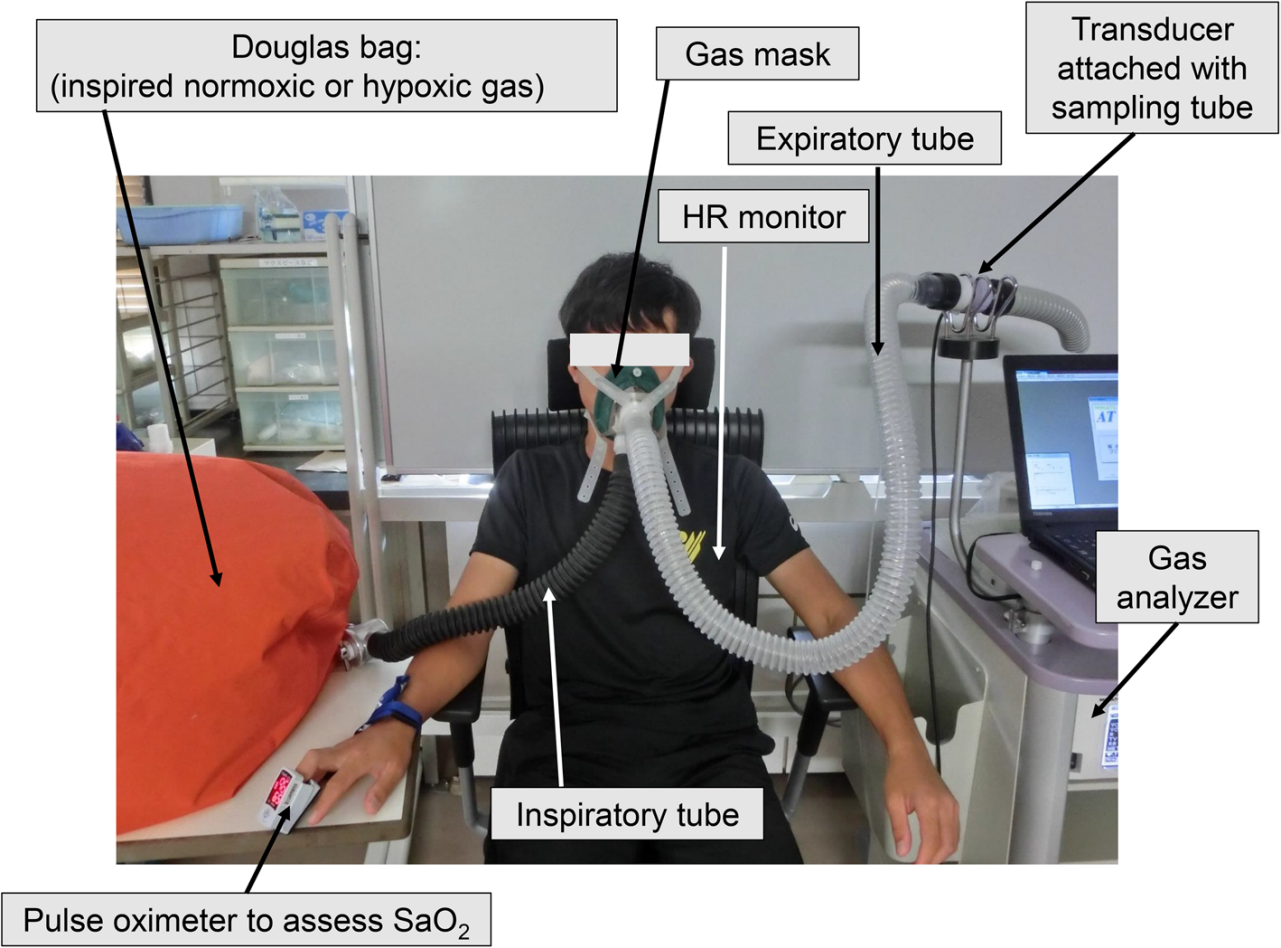
Experimental set up. HR, heart rate; SaO_2_, peripheral arterial oxygen saturation

For the progressive hypoxic exposure test, each participant sat on a comfortable chair in a semi-recumbent position. They breathed room air (21% O_2_), followed by three graded hypoxic gas mixtures containing 18, 15, and 12% O_2_ for 60 min each. The last 5 min during the first 30 min of exposure (i.e., 25–30 min during each exposure period) were recorded (Fig. 1). This is because it has been reported that ventilatory responses was blunted 30 min after hypoxic exposure [15], and other measurements were performed during the latter 30 min in the previous study [9]. The hypoxic gas was continuously blended using a gas mixing system (YHS-B05S; YKS, Nara, Japan) and delivered from a 200 L Douglas bag reservoir through a two-way, non-rebreathing valve and face mask (Fig. 2).

### Measurements

Pulmonary ventilation ($\dot{\mathrm V}$_E_; L min^−1^), $\dot{\mathrm V}$O_2_, and carbon dioxide output ($\dot{\mathrm V}$CO_2_) were continuously measured by an online computerized metabolic cart (aero monitor AE-300S, Minato Medical Science, Osaka, Japan). The $\dot{\mathrm V}$_E_, $\dot{\mathrm V}$O_2_, and $\dot{\mathrm V}$CO_2_ were averaged every 10 s. Heart rate (HR; beats min^−1^) and peripheral arterial oxygen saturation (SaO_2_; %) were monitored with a wireless HR monitor (POLAR RC800X, POLAR Electro, Tokyo, Japan) and finger pulse oximetry (PULSOX-300i, Konica Minolta, Tokyo, Japan), respectively. These data (HR and SpO_2_) were also averaged every 10 s.

### Data analysis

Based on previous studies [7, 16], the hypoxic desaturation (ΔSaO_2_), hypoxic ventilatory (HVR), and cardiac (HCR) responses were calculated by the following equations.

$$\Delta {\textrm{SaO}}_2=\left({\textrm{SaO}}_2\ \textrm{at}\ \textrm{normoxia}\right)-\left({\textrm{SaO}}_2\ \textrm{at}\ \textrm{each}\ \textrm{hypoxia}\ \left[18,15,\textrm{or}\ 12\%{\textrm{O}}_2\right]\right)\left(\%\right)$$

$$\textrm{HVR}=\left(\dot{\mathrm{V}}_{\textrm{E}}\ \textrm{at}\ \textrm{each}\ \textrm{hypoxia}-\dot{\mathrm{V}}_{\textrm{E}}\ \textrm{at}\ \textrm{normoxia}\right)/\left(\Delta {\textrm{SaO}}_2\ \textrm{at}\ \textrm{each}\ \textrm{hypoxia}\times \textrm{body}\ \textrm{weight}/100\right)\ \left(\ \textrm{L}\ {\min}^{-1}\ {\%}^{-1}\ {\textrm{kg}}^{-1}\right)$$

$$\textrm{HCR}=\left(\textrm{HR}\ \textrm{at}\ \textrm{each}\ \textrm{hypoxia}-\textrm{HR}\ \textrm{at}\ \textrm{normoxia}\right)/\Delta {\textrm{SaO}}_2\ \left(\textrm{beats}\ {\min}^{-1}\ {\%}^{-1}\right)$$

The HVR and HCR were calculated using data every 10 s (*see above*) and averaged over the 5-min period. Since ΔSaO_2_ is defined as the difference from normoxia to hypoxia, greater ΔSaO_2_ values indicate greater arterial desaturation. Accordingly, greater values of HVR or HCR mean more effective responses to hypoxia [7, 16]. Percent changes in $\dot{\mathrm V}$O_2peak_, HVR, and HCR was calculated by a following equation.

$$\textrm{Percent}\ \textrm{changes}\ \textrm{in}\ \textrm{these}\ \textrm{physiological}\ \textrm{variables}\ \left({\dot{\mathrm{V}}\mathrm{O}}_{2\textrm{peak}},\textrm{HVR},\textrm{and}\ \textrm{HCR}\right)=\left[\textrm{these}\ \textrm{variables}\ \textrm{at}\ \textrm{each}\ \textrm{oxygen}\ \textrm{level}\ \left(18\%,15\%,\textrm{or}\ 12\%\right)-\textrm{at}\ 21\%{\textrm{O}}_2\right]/ \ \textrm{these}\ \textrm{variables}\ \textrm{at}\ 21\%{\textrm{O}}_2\times 100\ \left(\%\right)\kern0.5em$$

### Statistical analysis

Values are mean ± standard deviation. A paired *t*-test was used for $\dot{\mathrm V}$O_2peak_ comparison between normoxia and hypoxia. One-way repeated-measures analysis of variance with pairwise (Bonferroni) post hoc tests were used to evaluate the changes in all cardiorespiratory variables ($\dot{\mathrm V}$_E_, HR, SaO_2_, SaO_2_, HVR, and HCR) across different oxygen levels. Effect size was calculated as “*η*^2^,” defined as small (*η*^2^ = 0.01), medium (*η*^2^ = 0.06), and large (*η*^2^ = 0.14) effects [17]. The Pearson correlation coefficient was used for the relationship between decreases in $\dot{\mathrm V}$O_2peak_ and changes in SaO_2_, HVR, and HCR at each level. When a significant correlation was found between variables, we performed multiple linear regression analysis using variables that was recognized a significant relationship to explain changes in $\dot{\mathrm V}$O_2peak_. Although high variance inflation factors (VIFs > 5) indicate multicollinearity [18], the VIFs were < 3 for all explanatory variables. To identify the optimal model containing the parameters to best explain the data, we performed model selection by backward stepwise elimination using Akaike Information Criterion based on our previous studies [19, 20]. All statistical analyses were performed using R ver. 2.13.1. A *P* value < 0.05 was considered statistically significant.

## Results

Table 1 showed cardio respiratory variables at exhaustion during an incremental exercise test.

**Table 1:** Cardiorespiratory variables at exhaustion during incremental ramp exercise test

	Normoxia	Hypoxia	*P* value
$\dot{\mathrm V}$O_2_, ml/min/kg	49.9±10.4	38.8±7.2	< 0.001
$\dot{\mathrm V}$CO_2_, ml/min/kg	52.4±10.2	42.1±7.5	< 0.001
$\dot{\mathrm V}$_E_, L/min	112.9±17.9	116.8±14.4	0.153
RER	1.05±0.06	1.09±0.09	0.258
HR, bpm	185±3	189±4	0.089

Values are mean ± standard deviation (SD). $\dot{\mathrm V}$*O*_*2*_, pulmonary oxygen uptake; $\dot{\mathrm V}$*CO*_*2*_, carbon dioxide output; $\dot{\mathrm V}$_*E*_, pulmonary ventilation; *RER*, respiratory gas exchange ratio; *HR*, heart rate; *bpm*, beats per minute

$\dot{\mathrm V}$O_2peak_ and $\dot{\mathrm V}$CO_2peak_ during the incremental leg cycling test decreased in hypoxia compared with that in normoxia (*P* < 0.001, respectively). The average decrease in $\dot{\mathrm V}$O_2peak_ was − 21.4 ± 8.7%. In contrast, no significant differences in $\dot{\mathrm V}$_E_ and respiratory gas exchange ratio between the normoxia and hypoxia. The HR in hypoxia was marginally lower compared to normoxia (*P* < 0.1).

**Fig. 3:**
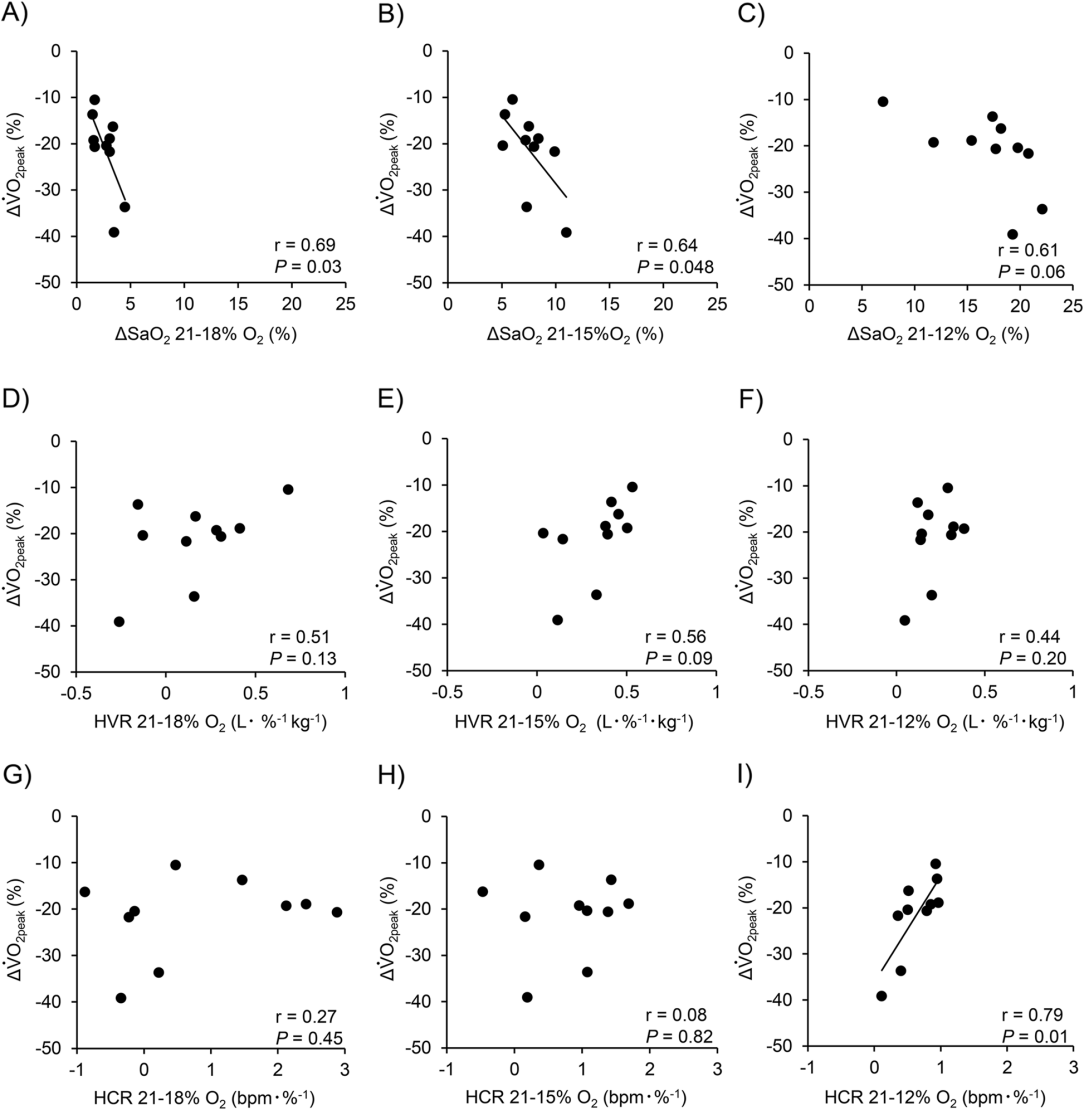
Relationships between changes in peak oxygen uptake ($\dot{\mathrm V}$O_2peak_) and arterial desaturation (ΔSaO_2_ [**A**–**C**]), hypoxic ventilatory and cardiac responses (HVR [**D**–**F**] and HCR [**G**–**I**]) at each oxygen level

Cardiorespiratory variables across the four O_2_ levels are shown in Table 2. $\dot{\mathrm V}$_E_ and HR progressively increased, while SaO_2_ progressively decreased. Below the 15% O_2_ level, there were significant differences in $\dot{\mathrm V}$_E_, HR, and SaO_2_ compared with those at 21% and 18% O_2_ (all *P* < 0.05, Table 2). Moreover, HR and SaO_2_ at 12% O_2_ were significantly higher and lower, respectively, compared with those at 15% O_2_ (*P* < 0.05). ΔSaO_2_ progressively increased from 18 to 12% O_2_, with significant differences between each oxygen level (all *P* < 0.05). HVR significantly increased from 18 to 15% O_2_, after which the value stabilized. No significant differences were observed in the HCR across the four O_2_ levels (all *P* > 0.05, Table 3). Figure 3 shows the relationships between oxygen level-induced changes in $\dot{\mathrm V}$O_2peak_ and in SaO_2_, HVR, and HCR. Changes in $\dot{\mathrm V}$O_2peak_ were associated with changes in SaO_2_ from 21 to 18 and 15% O_2_ and in the HCR from 21 to 12% O_2_ (Fig. 3A, B, and I, *P* < 0.05), while other cardiorespiratory variables were not associated with changes in $\dot{\mathrm V}$O_2peak_ (Fig. 3C–H, all *P* > 0.05). Relative changes in $\dot{\mathrm V}$O_2peak_ were explained by the following equation using multiple regression analysis:

$$\textrm{Changes}\ \textrm{in}\ {\dot{\mathrm{V}}\mathrm{O}}_{2\textrm{peak}}=-24.03+\left(18.60\times \textrm{HCR}\ \textrm{from}\ 21\ \textrm{to}\ 12\%{\textrm{O}}_2\right)+\left(-1.23\times {\textrm{SaO}}_2\ \textrm{from}\ 21\ \textrm{to}\ 15\%{\textrm{O}}_2\right); \ \left(\textrm{adjusted}\ {R}^2=0.580,P=0.02\right).\kern0.5em$$

**Table 2:** Cardiorespiratory variables during progressive hypoxia

	Inspired oxygen level				One-way ANOVA results		
	21% O_2_	18% O_2_	15% O_2_	12% O_2_	*F*	*P*	*η*^2^
$\dot{\mathrm V}$_E_, L min^−1^	9.6±1.4	9.9±1.5	11.3±1.6^a,b^	12.0±1.7^a,b^	40.3	< 0.001	0.32
HR, bpm	62.4±9.2	63.8±8.6	67.9±9.3^a,b^	72.4±9.7^a,b,c^	28.0	< 0.001	0.17
SaO_2_, %	97.0±0.6	94.3±1.0	89.4±1.9^a,b^	80.0±4.5^a,b,c^	107.0	< 0.001	0.88

Values are mean ± standard deviation (SD). $\dot{\mathrm V}$_*E*_ pulmonary ventilation, *HR* heart rate, *bpm* beats per minute, *SaO*_*2*_ arterial oxygen saturation

^a^, ^b^, or ^c^ indicate significant differences vs. 21%, 18% or 15% O_2_, respectively

**Table 3:** Cardiorespiratory variables during progressive hypoxia

	Differences between each oxygen level			One-way ANOVA results		
	From 21 to 18% O_2_	From 21 to 15% O_2_	From 21 to 12% O_2_	*F*	*P*	*η*^2^
ΔSaO_2_, %	2.7±1.0	7.6±1.9^a^	16.9±4.5^a,b^	84.7	< 0.001	0.82
HVR, L min^−1^ kg^−1^	0.16±0.29	0.33±0.17^a^	0.21±0.11	4.53	0.03	0.12
HCR, beats min^−1^ %^−1^	0.80±1.32	0.79±0.69	0.64±0.30	0.20	0.82	0.01

Values are mean ± SD. HVR, ventilatory response to hypoxia; HCR, cardiac response to hypoxia. ^a^ or ^b^ indicate significant differences vs. (21–18% O_2_) or (21–15% O_2_), respectively. Note that as ΔSaO_2_ was calculated as the difference from the values of normoxia to hypoxia, and therefore, greater plus values indicate greater desaturation

When using partial correlation coefficients, the contribution rate of each variable was 70.6% of HCR and 29.4% of SaO_2_ for changes in $\dot{\mathrm V}$O_2peak_.

## Discussion

We found that HCR and arterial desaturation, rather than HVR, may be associated with changes in $\dot{\mathrm V}$O_2peak_. Arterial desaturation increased with decreasing fractions of inspired O_2_. $\dot{\mathrm V}$_E_ and HR also increased, possibly due to stimulation of peripheral chemoreceptors [21], increased sympathetic activity, and vagal withdrawal [22].

HVR reportedly significantly increased with decreasing O_2_ levels (14.5–12.7–11.5% O_2_) [7], which was partly inconsistent with our findings. Additionally, we found no significant relationships between ΔHVR and Δ$\dot{\mathrm V}$O_2peak_ at all O_2_ levels (Fig. 3D–F), which contradicted a previous study [5]. These discrepancies may be related to HVR assessments across different time domains. In this study, the HVR at 12% O_2_ was assessed after 2 h 25 min exposure to hypoxia (Fig. 1), whereas a previous study assessed the HVR during the first 4 min of hypoxic exposure [7]. An initial rise in $\dot{\mathrm V}$_E_ was followed by a marked decline after several minutes to an intermediate value over 25 min of hypoxia [15, 23]. Thus, our longer-term measurements may cause underestimation of increases in $\dot{\mathrm V}$_E_ and HVR compared to previous studies [5, 7]. However, we found the HVR at 15% O_2_ was significantly greater than at 18% O_2_, suggesting light hypoxia at 18% O_2_ is not a sufficient stimulus to $\dot{\mathrm V}$_E_ responses compared with 15% O_2_ (Table 2).

It should be noted that greater individual variance of HCR, calculated by dividing ΔHR by ΔSaO_2_, was observed in the present study (Table 2 and Fig. 3). In hypoxia, previous studies demonstrated that changes in SaO_2_ were inversely associated with ratio of low-frequency to high-frequency (frequency domain of heart rate variability) [24] and proportionally associated with root mean square successive difference (time domain of heart rate variability) [25]. Thus, changes in HCR can be partly contributed to a balance of cardiac autonomic nervous activity. Importantly, interindividual variations of heart rate variability has been considerably greater [26], which might explain greater individual variance of HCR in the present study.

Another possible explanation may be arterial baroreflex function rather than pulmonary inflation of reflex, which is caused by vagal withdrawal to hypoxic exposure [27]. However, we must acknowledge that this hypothesis is speculative, and therefore, future studies should be warranted.

The ΔSaO_2_ in this study was greater than that found in a previous study [7] (i.e., 7.6% at 15% O_2_ and 16.9% at 12% O_2_ vs. 4.3% at 14.5% O_2_ and 9.9% at 11.7% O_2_). Additionally, unlike a previous study [7], we found no significant differences in the HCR across different hypoxia levels. As greater ΔSaO_2_ values lead to lower HCR, a greater reduction in SaO_2_ may have mitigated the increase in HCR despite the elevation of HR. Although the detailed physiological mechanisms of ΔSaO_2_ in hypoxia are uncertain, population features might be related with these inconsistencies. Specifically, the participants in the previous study had no history of acute mountain sickness, whereas about 60% of our participants experienced symptoms of AMS [9]. The degree of oxygen desaturation has been reported as an important predictor of acute mountain sickness [8, 16, 28–31].

Notably, we found significant relationships between Δ$\dot{\mathrm V}$O_2peak_ and hypoxic-induced cardiac responses (Fig. 3A, B, and I). Exercise-induced arterial hypoxemia influences the decline of $\dot{\mathrm V}$O_2max_ in mild hypoxia [32, 33]. Additionally, greater arterial desaturation and lower $\dot{\mathrm V}$O_2peak_ were reportedly observed in chronic obstructive pulmonary disease patients compared with those in healthy populations [34], suggesting a relation between arterial desaturation and aerobic capacity in healthy and diseases populations. The absence of a correlation between ΔSaO_2_ and Δ$\dot{\mathrm V}$O_2peak_ at 21–12% O_2_ in this study suggests elevated HR can compensate arterial desaturation (Fig. 3I).

To clarify the contributions of each index on the decline in $\dot{\mathrm V}$O_2peak_, the optimal model could reveal further insight. The model showed that the contribution rate of HCR to changes in $\dot{\mathrm V}$O_2peak_ is approximately 2.3 times than that of SaO_2_. This suggests that HR responses to greater arterial desaturation under severe hypoxia at rest play an important role in preventing declines in $\dot{\mathrm V}$O_2peak_ at moderate hypoxia. One study exhibited more than double circulatory (HR) than respiratory ($\dot{\mathrm V}$_E_) energy expenditure costs in hypoxia regardless of rest or exercise [35], which could support these findings.

### Study limitations and strengths

A major strength of this study is the relatively simple method using a multistep resting hypoxia test to assess the hypoxic exercise-induced declines in peak aerobic capacity. However, several limitations include small sample size, limited physiological variables as other possible predictors, and the retrospective analysis of data [9]. Second, we recruited only young men for the present study. Previous studies reported that sex- and age-related hypoxic ventilatory and/or cardiac responses were different between men and women [36, 37] or between young and old adults [36]. Thus, to generalize our results for wider population, future studies are warranted including women and/or aged people. Finally, during an incremental exercise test, we could not measure SaO_2_ that has been advocated as one potential candidate to decrease in $\dot{\mathrm V}$O_2peak_ in a previous study [32].

In conclusion, this study suggests that arterial desaturation at moderate hypoxia and cardiac responses at severe hypoxia may account for hypoxic-induced declines in peak aerobic capacity, and hypoxic ventilatory responses may be unrelated, at least, in the population of the present study.

## Data Availability

The data of this study are available from the corresponding author upon reasonable request.

## Abbreviations

ANOVA: Analysis of variance
FiO_2_: Fraction of inspired oxygen
HCR: Hypoxic cardiac response
HR: Heart rate
HVR: Hypoxic ventilatory response
SaO_2_: Peripheral arterial oxygen saturation
SD: Standard deviation
$$\dot{\mathrm V}$$_E_: Pulmonary ventilation
VIF: Variance inflation factor
$\dot{\mathrm V}$O_2peak_: Peak oxygen uptake

## References

[1] Fulco CS, Rock PB, Cymerman A (1998). Maximal and submaximal exercise performance at altitude. Aviat Space Environ Med.

[2] DeLorey DS, Shaw CN, Shoemaker JK, Kowalchuk JM, Paterson DH (2004). The effect of hypoxia on pulmonary O2 uptake, leg blood flow and muscle deoxygenation during single-leg knee-extension exercise. Exp Physiol.

[3] Horiuchi M, Handa-Kirihara Y, Abe D, Fukuoka Y (2019). Combined effects of exposure to hypoxia and cool on walking economy and muscle oxygenation profiles at tibialis anterior. J Sports Sci.

[4] Byrne-Quinn E, Weil JV, Sodal IE, Filley GF, Grover RF (1971). Ventilatory control in the athlete. J Appl Physiol.

[5] Ogawa T, Hayashi K, Ichinose M, Nishiyasu T (2007). Relationship between resting ventilatory chemosensitivity and maximal oxygen uptake in moderate hypobaric hypoxia. J Appl Physiol.

[6] Sheel AW, Koehle MS, Guenette JA, Foster GE, Sporer BC, Diep TT (2006). Human ventilatory responsiveness to hypoxia is unrelated to maximal aerobic capacity. J Appl Physiol.

[7] Lhuissier FJ, Brumm M, Ramier D, Richalet JP (2012). Ventilatory and cardiac responses to hypoxia at submaximal exercise are independent of altitude and exercise intensity. J Appl Physiol.

[8] Karinen HM, Peltonen JE, Kähönen M, Tikkanen HO (2010). Prediction of acute mountain sickness by monitoring arterial oxygen saturation during ascent. High Alt Med Biol.

[9] Horiuchi M, Endo J, Dobashi S, Kiuchi M, Koyama K, Subudhi AW (2016). Effect of progressive normobaric hypoxia on dynamic cerebral autoregulation. Exp Physiol.

[10] Foster GE, McKenzie DC, Milsom WK, Sheel AW (2005). Effects of two protocols of intermittent hypoxia on human ventilatory, cardiovascular and cerebral responses to hypoxia. J Physiol.

[11] Chapman RF, Stray-Gundersen J, Levine BD (1998). Individual variation in response to altitude training. J Appl Physiol.

[12] Roach RC, Saltin B, Boushel R, Secher N, Mitchell H (2000). Cardiovascular regulation during hypoxia. Exercise and circulation in health and disease.

[13] Wilber RL (2007). Application of altitude/hypoxic training by elite athletes. Med Sci Sports Exerc.

[14] Dobashi S, Horiuchi M, Endo J, Kiuchi M, Koyama K (2016). Cognitive function and cerebral oxygenation during prolonged exercise under hypoxia in healthy young males. High Alt Med Biol.

[15] Easton PA, Slykerman LJ, Anthonisen NR (1986). Ventilatory response to sustained hypoxia in normal adults. J Appl Physiol.

[16] Richalet JP, Larmignat P, Poitrine E, Letournel M, Canoui-Poitrine F (2012). Physiological risk factors for severe high-altitude illness: a prospective cohort study. Am J Respir Crit Care Med.

[17] Lakens D (2013). Calculating and reporting effect sizes to facilitate cumulative science: a practical primer for t-tests and ANOVAs. Front Psychol.

[18] Zuur AF, Ieno EN, Smith GM, Zuur AF, Ieno EN, Smith GM (2007). Monitoring for change: Using generalized least squares, non-metric multidimensional scaling, and Mantel test on western Montana grasslands. Analysing Ecological Data.

[19] Horiuchi M, Watanabe M, Mitsui S, Uno T (2021). Does change in barometric pressure per given time at high altitude influence symptoms of acute mountain sickness on Mount Fuji? A pilot study. J Physiol Anthropol.

[20] Uno T, Fujino M, Ohwaki A, Horiuchi M (2019). Prevalence of falls on Mount Fuji and associated with risk factors: a questionnaire survey study. Int J Environ Res Public Health.

[21] Heistad DD, Abboud FM, Dickinson W (1980). Richards lecture: circulatory adjustments to hypoxia. Circulation..

[22] Koller EA, Drechsel S, Hess T, Macherel P, Boutellier U (1988). Effects of atropine and propranolol on the respiratory, circulatory, and ECG responses to high altitude in man. Eur J Appl Physiol Occup Physiol.

[23] Morrill CG, Meyer JR, Weil JV (1975). Hypoxic ventilatory depression in dogs. J Appl Physiol.

[24] Krejčí J, Botek M, McKune AJ (2018). Dynamics of the heart rate variability and oxygen saturation response to acute normobaric hypoxia within the first 10 min of exposure. Clin Physiol Funct Imaging.

[25] Botek M, Krejčí J, De Smet S, Gába A, McKune AJ (2015). Heart rate variability and arterial oxygen saturation response during extreme normobaric hypoxia. Auton Neurosci.

[26] Kobayashi H (2007). Inter- and intra-individual variations of heart rate variability in Japanese males. J Physiol Anthropol.

[27] Siebenmann C, Ryrsø CK, Oberholzer L, Fisher JP, Hilsted LM, Rasmussen P (2019). Hypoxia-induced vagal withdrawal is independent of the hypoxic ventilatory response in men. J Appl Physiol.

[28] Burtscher M, Flatz M, Faulhaber M (2004). Prediction of susceptibility to acute mountain sickness by SaO_2_ values during short-term exposure to hypoxia. High Alt Med Biol.

[29] Burtscher M, Philadelphy M, Gatterer H, Burtscher J, Faulhaber M, Nachbauer W (2019). Physiological responses in humans acutely exposed to high altitude (3480 m): minute ventilation and oxygenation are predictive for the development of acute mountain sickness. High Alt Med Biol.

[30] Faulhaber M, Wille M, Gatterer H, Heinrich D, Burtscher M (2014). Resting arterial oxygen saturation and breathing frequency as predictors for acute mountain sickness development: a prospective cohort study. Sleep Breath.

[31] Roach RC, Greene ER, Schoene RB, Hackett PH (1998). Arterial oxygen saturation for prediction of acute mountain sickness. Aviat Space Environ Med.

[32] Chapman RF, Emery M, Stager JM (1999). Degree of arterial desaturation in normoxia influences VO2max decline in mild hypoxia. Med Sci Sports Exerc.

[33] Gaston AF, Durand F, Roca E, Doucende G, Hapkova I, Subirats E (2016). Exercise-induced hypoxaemia developed at sea-level influences responses to exercise at moderate altitude. PLoS One.

[34] Gonzalez-Garcia M, Barrero M, Maldonado D (2021). Exercise Capacity, Ventilatory response, and gas exchange in COPD patients with mild to severe obstruction residing at high altitude. Front Physiol.

[35] Horiuchi M, Fukuoka Y, Handa Y, Abe D, Pontzer H (2017). Measuring the energy of ventilation and circulation during human walking using induced hypoxia. Sci Rep.

[36] Camacho-Cardenosa A, Camacho-Cardenosa M, Tomas-Carus P, Timón R, Olcina G, Burtscher M (2022). Acute physiological response to a normobaric hypoxic exposure: sex differences. Int J Biometeorol.

[37] Richalet JP, Lhuissier F, Jean D (2020). Ventilatory response to hypoxia and tolerance to high altitude in women: influence of menstrual cycle, oral contraception, and menopause. High Alt Med Biol.

